# Prevalence of Depression Among the Adult Population in Southwestern Saudi Arabia: A Cross-Sectional, Community-Based Study

**DOI:** 10.7759/cureus.56081

**Published:** 2024-03-13

**Authors:** Mosad Odah, Ashraf Ewis, Fuad M Alkudaysi, Turki Alhasani, Awad A Alessi, Yahya Mohammed Y AlKudaysi, Khalid Abdullah M Alfaqih, Mohammed Ali O Alfaqih, Abdulaziz A Almatrafi, Amjad Z Nmnkani, Amirah S Alzubaidi, Atheer O Alothman, Amer S Alnashri, Maram M Almathami

**Affiliations:** 1 Biochemistry, Umm Al-Qura University, Al-Qunfudah, SAU; 2 Public Health, Umm Al-Qura University, Al-Qunfudah, SAU; 3 Pediatrics, South Al-Qunfudah General Hospital, Al-Qunfudah, SAU; 4 Pediatrics, Khamis Mushayt Maternity and Children Hospital, Al-Qunfudah, SAU; 5 General Practice, Al-Sharqia Primary Health Center, Ministry of Health, Al-Qunfudah, SAU; 6 Medicine and Surgery, Umm Al-Qura University, Al-Qunfudah, SAU

**Keywords:** phq-9, saudi arabia, al-qunfudah, depression, adult, prevalence

## Abstract

Background

Depression is one of the most common mental disorders, which is increasing globally with higher prevalence among women. Many factors contribute to the etiology and risk factors for depression, including biological and psychosocial factors. This study aimed to assess the prevalence of depression among the adult population in Al-Qunfudah governorate, southwestern Saudi Arabia (SA).

Methods

A cross-sectional study was conducted on a sample of 1036 participants among adults in Al-Qunfudah governorate, southwestern SA, using a validated Arabic version of the Patient Health Questionnaire (PHQ-9) during the period from October 1st, 2022 to the end of December 2022. The PHQ-9 contains nine items, with a total score ranging from 0 to 27. A score of 1-4 represented minimal depression, while a score of 5, 10, 15, and 20 represented mild, moderate, moderately severe, and severe depression, respectively. The sample size was estimated to be 375 participants, by considering a margin of error of 5%, and a 95% confidence interval, calculated using Raosoft calculator (Raosoft Inc., Seattle, WA). Data collection was performed through an online survey of the PHQ-9 on a Google form and distributed using different social media platforms. The eligible participants' responses were kept confidential and analyzed using IBM SPSS Statistics for Windows, Version 22 (Released 2013; IBM Corp., Armonk, New York, United States). p-values of <0.05 were considered statistically significant.

Results

The study showed that the overall prevalence of depression among the 1036 adult study participants was 68.1%. Mild, moderate, moderate to severe, and severe depression was diagnosed among 28.2%, 21.9%, 12%, and 6% of the participants, respectively. Several factors were significantly associated with PHQ-9 diagnosed depression including being younger (p<0.0001), a female (p<0.0001), single (p<0.0001), a student (p<0.0001), and non-employed (p<0.0001) and having a lower educational level (p<0.0001).

Conclusions

There is a high prevalence rate of depression among the adult population of Al-Qunfudah governorate in southwestern SA, which highlights the need for interventions to address this issue, and to reduce the incidence of depression in the region among the high-risk groups.

## Introduction

A state of complete mental well-being is a part of the World Health Organization (WHO) health definition [1]. A mental disorder is a clinically significant disturbance of cognition, emotions, or behaviors of the patient that interferes with normal personal or social activities and causes a significant disability or even mortality [2,3]. Many adverse events in life could increase the risk for mental disorders including poverty, violence, and disability. On the other hand, many people with mental disorders lack proper health care access, due to various reasons. The coronavirus disease 2019 (COVID-19) pandemic for instance has significantly affected many people with anxiety and depression [4]. The COVID-19 pandemic has had an impact on mental health, and the prevalence of mental health problems including depression increased during this pandemic [5].

Depression is a major leading cause of disability and significantly causes a global burden [6]. According to the WHO, 280 million around the world have had depression in 2019 [4]. Furthermore, it was estimated that the global prevalence of depression is 3.8%, which includes 5% in adults and 5.7% in those older than 60 years, and it affects women predominantly [6]. In Saudi Arabia (SA), several studies have been conducted in different regions indicating prevalence rates of depression that ranged between 8.6% and 49.9% [7-10]. 

Depression is characterized by symptoms of depressed mood, loss of interest, and other disabling symptoms such as insomnia and fatigue [2]. Many factors contribute to depression etiology and risk factors including biological factors and psychosocial factors [11]. For the social factors, a higher incidence of major depressive disorder (MDD) was reported among women compared to men, while about half of MDD onset started between 20 and 50 years of age. Furthermore, MDD was more frequent among people who are divorced, separated, and who do not have a close interpersonal relationship. Psychosocial risk factors include life events and environmental stressors. On the contrary, there is a poor association between MDD incidence and socioeconomic status or educational level. People who suffer from comorbidities such as alcohol abuse or dependence, anxiety disorders, and other mood disorders, are at high risk for MDD also. Although the biological process and pathogenesis of MDD are still unclear, many theories suggest neurological, hormonal, immunological, and genetic involvement [12].

Since depression is an underdiagnosed disorder [13], with significant individual and economic impacts [14,15], there is great importance in identifying its leading risk factors and the high-risk groups, while acting properly in order to reduce its incidence [16]. However, there are no sufficient data on the prevalence of depression in the southwestern region of SA especially in the Al-Qunfudah governorate. Therefore, this study aimed at assessing the prevalence of depression among the adult population in Al-Qunfudah governorate, SA.

## Materials and methods

Study design

A descriptive cross-sectional, community-based study was executed among adults aged 18 and above in Al-Qunfudah governorate, southwestern Saudi Arabia.

Ethical considerations

Ethical approval was sought from Umm Al-Qura IRB NO. (HAPO-02-K-012-2022-11-1318). Consent was obtained from all participants before the questionnaire was filled. No personal identifying data were collected from participants and all data were coded and handled carefully to ensure its safety and confidentiality.

Study population and setting

The study population included male and female adults aged 18 years and above who lived in the Al-Qunfudah governorate and agreed to participate in this study voluntarily, after being assured that all data would be collected anonymously and kept confidential. Responders whose age was less than 18 years or were residents of other areas than Al-Qunfudah were excluded from the analysis.

The study was conducted among the adult population of Al-Qunfudah, a southwestern governorate, which is located on the Red Sea coast, south of Jeddah city, Makkah Province, SA.

Study procedures & data collection

A validated Arabic version of the Patient Health Questionnaire (PHQ-9) [17,18], was used to assess depressive symptoms, after taking permission from the corresponding author. The PHQ-9 contains nine items, each of which is scored from 0 (never), 1 (present in several days), 2 (present more than half the days), to 3 (almost every day) to produce a total score ranging from 0 to 27. The diagnosis of depression was performed based on DSM-5 diagnostic criteria for major depressive disorder. Scores of 1-4 represented minimal depression, while a score of 5, 10, 15, and 20 represented mild, moderate, moderately severe, and severe depression, respectively. For statistical analysis in this study, subjects with minimal scores 1-4 on PHQ-9, were considered not to have depression. PHQ-9 is a valid brief depression severity measure, with a sensitivity of 88% and a specificity of 88% for major depression with a score of ≥10. Using this tool, major depression can be diagnosed with a score of ≥5 [19]. Sociodemographic data were also collected including age, sex, nationality, residency, marital status, occupational status, and educational level.

The data collection was performed over a duration of three months starting from the beginning of October 2022 to the end of December 2022 through an online survey that was designed on a Google form and distributed using different social media platforms.

Sample size calculation

The sample size was calculated using the Raosoft calculator (Raosoft Inc., Seattle, WA) using the Equation n=72 x p (1-p)/e2 for which n is the sample size, z (1.96) is the z-score associated with a level of confidence (95%), p is the sample proportion and is expressed as a decimal, and e (0.05) is the margin of error expressed as a decimal. The calculated sample size was at least 375 participants. However, the data collectors received a total of 1036 valid responses, which is nearly 3-fold the estimated sample size. This number of participants could be beneficial to improve the statistical inferences and to overcome the over-representation of specific traits that could arise from the snowball sampling technique of the online data collection procedures. Additionally, a large sample size can improve the generalization of the results that are based on a wide range of data. 

Statistical analysis

The data were analyzed using IBM SPSS Statistics for Windows, Version 22 (Released 2013; IBM Corp., Armonk, New York, United States). Descriptive statistics were used to describe the study population. Quantitative data were expressed as means + standard deviations (SD) while categorical data were presented as numbers and percentages. The Chi-squared test was used to assess the association between categorical variables and PHQ-9 diagnosis. Pearson correlation coefficient testing (r) was performed to describe the association between the PHQ-9 score and the age of the study participants. p-values of <0.05 were considered statistically significant. 

## Results

The study included a total of 1036 participants. The sample is mostly young, with 569 (54.9%) of participants falling in the 21-30 age. There is a slightly higher proportion of females in the sample 530 (51.2%), and the majority of participants are Saudi citizens 1017 (98.2%). The sample is also predominantly single 655 (63.2%), with students making up the largest occupational group 499 (48.2%). The majority of participants have at least a university education 616 (59.5%) (Table 1).

**Table 1:** Sociodemographic characters of the participants (n=1036).

Parameter		Frequency (%)
Age, y	18 – 20	207 (20%)
	21 – 30	569 (54.9%)
	31 - 40	150 (14.5%)
	41 - 70	110 (10.6%)
Sex	Female	530 (51.2%)
	Male	506 (48.8%)
Nationality	Saudi	1017 (98.2%)
	Non-Saudi	19 (1.8%)
Residency	Ahad Bani zaid	9 (0.9%)
	Al-airdiat	19 (1.8%)
	Al-Qunfudah city	344 (33.2%)
	AlQuz	201 (19.4%)
	AlMuzailif	48 (4.6%)
	Hali	306 (29.5%)
	Khmais Harb	31 (3%)
	Doqa	78 (7.5%)
Marital status	Single	655 (63.2%)
	Married	349 (33.7%)
	Divorced & Widowed	32 (3.1%)
Occupational status	Student	499 (48.2%)
	Non-occupied	131 (12.6%)
	Occupied	406 (39.2%)
Educational level	Primary education	8 (0.8%)
	Intermediate education	30 (2.9%)
	Secondary education	241 (23.3%)
	Diploma	84 (8.1%)
	University	616 (59.5%)
	Master degree	38 (3.7%)
	PhD	19 (1.8%)

In this study, the responses to the nine questions of the PHQ-9 for the study sample were assessed, with the frequency and percentage of participants who reported each level of severity for each PHQ-9 question. Each question asks about the frequency of specific symptoms of depression over the past two weeks, and the responses are divided into four categories: "not at all", "several days", "more than half the days", and "nearly every day". For example, 407 (39.3%) of participants reported "not at all" experiencing "little interest or pleasure in doing things", while 197 (19%) reported experiencing this symptom "several days" in the past two weeks (Table 2).

**Table 2:** PHQ-9 Items and their responses (n=1036). PHQ-9: Patient Health Questionnaire

Item	Not at all	Several days	More than half the days	Nearly every day
Little interest or pleasure in doing things?	407 (39.3%)	197 (19%)	343 (33.1%)	89 (8.6%)
Feeling down, depressed, or hopeless?	328 (31.7%)	200 (19.3%)	406 (39.2%)	102 (9.8%)
Trouble falling or staying asleep, or sleeping too much?	361 (34.8%)	195 (18.8%)	315 (30.4%)	165 (15.9%)
Feeling tired or having little energy?	260 (25.1%)	241 (23.3%)	387 (37.4%)	148 (14.3%)
Poor appetite or overeating?	360 (34.7%)	223 (21.5%)	319 (30.8%)	134 (12.9%)
Feeling bad about yourself - or that you are a failure or have let yourself or your family down?	506 (48.8%)	158 (15.3%)	260 (25.1%)	112 (10.8%)
Trouble concentrating on things, such as reading the newspaper or watching television?	439 (42.4%)	170 (16.4%)	324 (31.3%)	103 (9.9%)
Moving or speaking so slowly that other people could have noticed? Or the opposite - being so fidgety or restless that you have been moving around a lot more than usual?	544 (52.5%)	127 (12.3%)	294 (28.4%)	71 (6.9%)
Thoughts that you would be better off dead, or of hurting yourself in some way?	682 (65.8%)	113 (10.9%)	180 (17.4%)	61 (5.9%)

The results of the PHQ-9 scores for the study sample were divided into diagnostic categories based on the total score. The diagnostic categories are "minimal depression", "mild depression", "moderate depression", "moderately severe depression", and "severe depression". The sample has a relatively high mean PHQ-9 score of 8, with a standard deviation of 6.3. This suggests that there is a significant amount of variability in the scores, with some participants scoring very low and others scoring very high. Based on the diagnostic categories, 330 (31.9%) of the sample falls into the "minimal depression" category, while 292 (28.2%) falls into the "mild depression" category. A total of 227 (21.9%) falls into the "moderate depression" category, 124 (12%) falls into the "moderately severe depression" category, and 64 (6.1%) falls into the "severe depression" category. Among the studied population, the prevalence of screened depression found to be 706 (68.1%) based on the predetermined cut-off limits on screening instruments (Table 3).

**Table 3:** PHQ-9 Score and diagnostic categories (n=1036). PHQ-9: Patient Health Questionnaire

Parameter		Frequency (%)
PHQ-9 Diagnosis (5 category scale)	Minimal depression	330 (31.9%)
	Mild depression	292 (28.2%)
	Moderate depression	227 (21.9%)
	Moderately severe depression	124 (12%)
	Severe depression	63 (6.1%)
PHQ-9 Diagnosis (2 category scale)	Absent	330 (31.9%)
	Present	706 (68.1%)
PHQ-9 Score	Mean±SD	8±6.3

**Figure 1:**
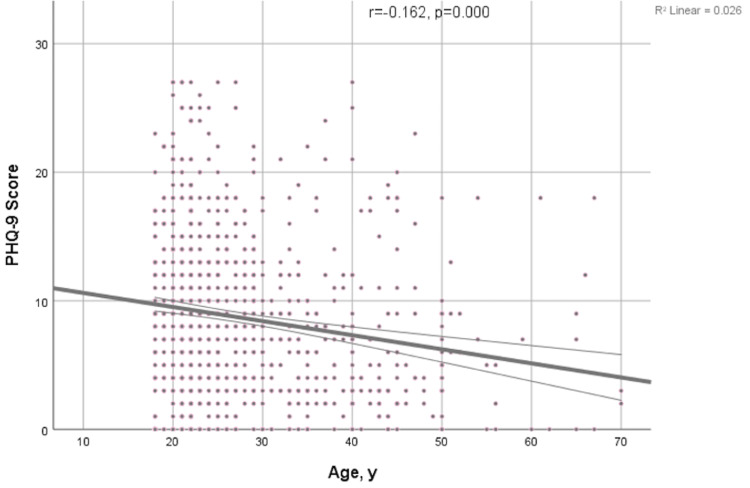
Scatter plot with line of PHQ-9 score and age (years); Pearson's correlation coefficient (r) and p-values are presented. PHQ-9: Patient Health Questionnaire

Several factors are significantly associated with PHQ-9 diagnosis and were assessed for association with the 5-category scale PHQ-9 diagnosis. Age for instance is significantly associated with PHQ-9 diagnosis (p<0.0001), with younger participants being more likely to have higher PHQ-9 scores (Figure 1).

Other factors include sex (p<0.0001), with females being more likely to have higher scores, marital status (p<0.0001), with single participants being more likely to have higher scores, occupational status (p<0.0001), with students and non-occupied participants being more likely to have higher scores, and educational level (p<0.0001), with those with higher levels of education being less likely to have higher scores (Table 4).

**Table 4:** Factors associated with PHQ-9 diagnosis (5-category scale) (n=1036). PHQ-9: Patient Health Questionnaire A p-value of <0.05 was considered statistically significant.

Parameter		Minimal depression	Mild depression	Moderate depression	Moderately severe depression	Severe depression	p-value
Age, y	18 – 20	62 (30%)	51 (24.6%)	49 (23.7%)	26 (12.6%)	19 (9.2%)	<0.0001
	21 – 30	147 (25.8%)	162 (28.5%)	147 (25.8%)	78 (13.7%)	35 (6.2%)	
	31 - 40	66 (44%)	49 (32.7%)	23 (15.3%)	5 (3.3%)	7 (4.7%)	
	41 - 70	55 (50%)	30 (27.3%)	8 (7.3%)	15 (13.6%)	2 (1.8%)	
Sex	Female	130 (24.5%)	154 (29.1%)	144 (27.2%)	57 (10.8%)	45 (8.5%)	<0.0001
	Male	200 (39.5%)	138 (27.3%)	83 (16.4%)	67 (13.2%)	18 (3.6%)	
Nationality	Saudi	323 (31.8%)	287 (28.2%)	224 (22%)	121 (11.9%)	62 (6.1%)	0.943
	Non-Saudi	7 (36.8%)	5 (26.3%)	3 (15.8%)	3 (15.8%)	1 (5.3%)	
Residency	Ahad Bani zaid	2 (22.2%)	2 (22.2%)	2 (22.2%)	2 (22.2%)	1 (11.1%)	0.078
	Al-airdiat	6 (31.6%)	8 (42.1%)	2 (10.5%)	2 (10.5%)	1 (5.3%)	
	Al-Qunfudah city	89 (25.9%)	100 (29.1%)	84 (24.4%)	41 (11.9%)	30 (8.7%)	
	AlQuz	53 (26.4%)	58 (28.9%)	44 (21.9%)	34 (16.9%)	12 (6%)	
	AlMuzailif	16 (33.3%)	14 (29.2%)	11 (22.9%)	5 (10.4%)	2 (4.2%)	
	Hali	124 (40.5%)	81 (26.5%)	57 (18.6%)	30 (9.8%)	14 (4.6%)	
	Khmais Harb	9 (29%)	7 (22.6%)	11 (35.5%)	4 (12.9%)	0 (0%)	
	Doqa	31 (39.7%)	22 (28.2%)	16 (20.5%)	6 (7.7%)	3 (3.8%)	
Marital status	Single	172 (26.3%)	175 (26.7%)	176 (26.9%)	80 (12.2%)	52 (7.9%)	<0.0001
	Married	145 (41.5%)	106 (30.4%)	46 (13.2%)	42 (12%)	10 (2.9%)	
	Divorced & Widowed	13 (40.6%)	11 (34.4%)	5 (15.7%)	2 (6.2%)	1 (3.1%)	
Occupational status	Student	131 (26.3%)	135 (27.1%)	121 (24.2%)	73 (14.6%)	39 (7.8%)	<0.0001
	Non-occupied	39 (29.8%)	47 (35.9%)	24 (18.3%)	13 (9.9%)	8 (6.1%)	
	Occupied	160 (39.4%)	110 (27.1%)	82 (20.2%)	38 (9.4%)	16 (3.9%)	
Educational level	Primary education	2 (25%)	2 (25%)	1 (12.5%)	3 (37.5%)	0 (0%)	<0.0001
	Intermediateeducation	7 (23.3%)	1 (3.3%)	4 (13.3%)	18 (60%)	0 (0%)	
	Secondaryeducation	97 (40.2%)	67 (27.8%)	45 (18.7%)	21 (8.7%)	11 (4.6%)	
	Diploma	38 (45.2%)	22 (26.2%)	11 (13.1%)	9 (10.7%)	4 (4.8%)	
	University	166 (26.9%)	186 (30.2%)	149 (24.2%)	68 (11%)	47 (7.6%)	
	Master degree	17 (44.7%)	11 (28.9%)	6 (15.8%)	4 (10.5%)	0 (0%)	
	PhD	3 (15.8%)	3 (15.8%)	11 (57.9%)	1 (5.3%)	1 (5.3%)	
Chi-Squared test was used.							

The previous factors also were assessed for association with the 2-category scale PHQ-9 diagnosis (Table 5).

**Table 5:** Factors associated with PHQ-9 diagnosis (2-category scale) (n=1036). PHQ-9: Patient Health Questionnaire A p-value of <0.05 was considered statistically significant.

Parameter		Absent	Present	p-value
Age, y	18 – 20	62 (30%)	145 (70%)	<0.0001
	21 – 30	147 (25.8%)	422 (74.2%)	
	31 - 40	66 (44%)	84 (56%)	
	41 - 70	55 (50%)	55 (50%)	
Sex	Female	130 (24.5%)	400 (75.5%)	<0.0001
	Male	200 (39.5%)	306 (60.5%)	
Nationality	Saudi	323 (31.8%)	694 (68.2%)	0.638
	Non-Saudi	7 (36.8%)	12 (63.2%)	
Residency	Ahad Bani zaid	2 (22.2%)	7 (77.8%)	0.003
	Al-airdiat	6 (31.6%)	13 (68.4%)	
	Al-Qunfudah city	89 (25.9%)	255 (74.1%)	
	AlQuz	53 (26.4%)	148 (73.6%)	
	AlMuzailif	16 (33.3%)	32 (66.7%)	
	Hali	124 (40.5%)	182 (59.5%)	
	Khmais Harb	9 (29%)	22 (71%)	
	Doqa	31 (39.7%)	47 (60.3%)	
Marital status	Single	172 (26.3%)	483 (73.7%)	<0.0001
	Married	145 (41.5%)	204 (58.5%)	
	Divorced & Widowed	13 (40.6%)	19 (59.4%)	
Occupational status	Student	131 (26.3%)	368 (73.7%)	<0.0001
	Non-occupied	39 (29.8%)	92 (70.2%)	
	Occupied	160 (39.4%)	246 (60.6%)	
Educational level	Primary education	2 (25%)	6 (75%)	<0.0001
	University	166 (26.9%)	450 (73.1%)	
	Secondary education	97 (40.2%)	144 (59.8%)	
	Diploma	38 (45.2%)	46 (54.8%)	
	PhD	3 (15.8%)	16 (84.2%)	
	Master degree	17 (44.7%)	21 (55.3%)	
	Intermediate education	7 (23.3%)	23 (76.7%)	

## Discussion

The aim of the present study was to determine the prevalence of depression among the adult population in this region using the PHQ-9 questionnaire. The results of the study showed that the mean PHQ-9 score among participants was 8±6.3, indicating the presence of moderate depression. Out of the screened 1036 participants, the overall prevalence of depression was found to be 68.1%. This prevalence rate appears to be higher than that of other recent studies conducted in other regions of SA. As a review of the literature showed, 49.9% of screened subjects revealed depressive symptoms in Riyadh [10], while the prevalence of depression was 27% in the Asir region [8]. Another study showed that 12% of the screened subjects were diagnosed with depression in southeastern SA [9]. Furthermore, the prevalence of MDD was 8.6% in Al-Ahsa [7]. While these previous studies have identified risk factors for depression, including biological and psychosocial factors, there is currently a lack of information on the prevalence of depression in the Al-Qunfudah governorate in SA [7-10]. However, the timing of conducting this study is in the year 2022, during the COVID-19 pandemic. This could provide a rationale reason to explain this high prevalence found among the adult participants from Al-Qunfudah. The COVID-19 pandemic itself has had a significant impact on patients with depression [4], but the prevalence of depression also increased during the pandemic period [5].

The present study shows a significant association between age and PHQ-9 diagnosis, with younger participants being more likely to have higher PHQ-9 scores, with the highest rates found among those aged 21-30. Despite the significant impact of depression [14,15] at any age [12], it remains an underdiagnosed disorder [13], which highlights the importance of identifying high-risk groups and taking action to reduce the incidence of depression [16]. Although some previous studies failed to link age and depression prevalence [7,10], a strong association between age and MDD has been reported, with onset starting between 20 and 50 [12]. In addition to that, the vast majority of the participants in this study were between 18 and 31 of age, which also could explain the high prevalence among younger participants in this study.

In this study, the participants who were single more likely to be diagnosed with depression. Compared to some of the previous studies that found no significant association between depression and marital status [7], it has been linked between personal relationships and depression, especially among divorced and separated people as reviewed by the literature [12,20].

Overall, the results of this study suggest that there is a high prevalence of depression among the adult population in the Al-Qunfudah governorate. Further research is needed to understand the factors contributing to this high rate and to develop interventions to address the issue. It is important for healthcare professionals and policymakers to be aware of the high prevalence of depression in this region and to take steps to address it. Additionally, the prevalence of depression among those with lower quality of life needs to be investigated also, as it could underscore the importance of addressing social determinants of health in order to improve mental health outcomes.

Limitations

This study has some limitations that should be acknowledged. The data collection method was restricted to the online method as the data collection period coincided with the COVID-19 pandemic in SA. This could carry a risk for non-response bias that could undermine the generalization of the study findings since the non-respondents might carry different characteristics compared to the respondents. To overcome the impact of this bias, we tried our best to give the maximum number of people among the target population access to the PHQ-9 questionnaire by forwarding the link of the online Google Form via different social platforms and by extending the data collection period to give as many respondents as possible the opportunity to participate in the survey.

## Conclusions

In conclusion, this study showed a high prevalence rate of depression among the studied population of Al-Qunfudah governorate, SA, according to the screening instrument used (PHQ-9). Being younger, female, and single, having a lower level of education, and being non-occupied and a student are the main factors significantly associated with diagnosis of depression.

Further research is needed to explore other specific factors contributing to the high prevalence of depression in Al-Qunfudah governorate. Additionally, the high prevalence of depression found in this study highlights the need for effective interventions to address this issue in the Al-Qunfudah governorate. Targeted interventions aimed at improving the quality of life, and increasing educational attainment, may be effective in reducing the burden of depression in this region.

## References

[1] (1946). Constitution of the World Health Organization. https://www.who.int/about/governance/constitution.

[2] Black DW, Grant JE (2014). DSM-5® Guidebook: The Essential Companion to the Diagnostic and Statistical Manual of Mental Disorders. https://psycnet.apa.org/record/2014-12601-000.

[3] Walker ER, McGee RE, Druss BG (2015). Mortality in mental disorders and global disease burden implications: a systematic review and meta-analysis. JAMA Psychiatry.

[4] (2022). Mental disorders. https://www.who.int/news-room/fact-sheets/detail/mental-disorders.

[5] Lakhan R, Agrawal A, Sharma M (2020). Prevalence of depression, anxiety, and stress during COVID-19 pandemic. J Neurosci Rural Pract.

[6] (62021). Depressive disorder (depression). https://www.who.int/news-room/fact-sheets/detail/depression.

[7] Al Rashed AS, Al-Naim AF, Almulhim BJ (2019). Prevalence and associated factors of depression among general population in Al-Ahsa, Kingdom of Saudi Arabia: a community-based survey. Neurol Psychiatry Brain Res.

[8] Alqahtani MM, Salmon P (2008). Prevalence of somatization and minor psychiatric morbidity in primary healthcare in Saudi Arabia: a preliminary study in Asir region. J Family Community Med.

[9] Abdelwahid Abdelwahid, H. A., & Al-Shahrani, S. I. (2011). Screening of depression among patients in family medicine in Southeastern. Saudi Arabia. Saudi Med J.

[10] Al-Qadhi W, Ur Rahman S, Ferwana MS, Abdulmajeed IA (2014). Adult depression screening in Saudi primary care: prevalence, instrument and cost. BMC Psychiatry.

[11] Sadock BJ (2015). Kaplan & Sadock's Synopsis of Psychiatry: Behavioral Sciences/Clinical Psychiatry.

[12] Jeon SW, Amidfar M, Kim YK (2017). Bio-psycho-social risk factors for depression. Major Depressive Disorder: Risk Factors, Characteristics and Treatment Options.

[13] Sheehan DV (2004). Depression: underdiagnosed, undertreated, underappreciated. Managed care (Langhorne).

[14] Tse WS, Bond AJ (2004). The impact of depression on social skills. J Nerv Ment Dis.

[15] Gelenberg AJ (2010). The prevalence and impact of depression. J Clin Psychiatr.

[16] Muñoz RF, Beardslee WR, Leykin Y (2012). Major depression can be prevented. Am Psychol.

[17] AlHadi AN, AlAteeq DA, Al-Sharif E (2017). An arabic translation, reliability, and validation of Patient Health Questionnaire in a Saudi sample. Ann Gen Psychiatry.

[18] Becker S, Al Zaid K, Al Faris E (2002). Screening for somatization and depression in Saudi Arabia: a validation study of the Phq in primary care. Int J Psychiatr Med.

[19] Kroenke K, Spitzer RL, Williams JB (2001). The PHQ-9: validity of a brief depression severity measure. J Gen Intern Med.

[20] Maurer DM (2012). Screening for depression. Am Fam Physician.

